# “I am going out!” – lifestyle sports and physical activity in adolescents

**DOI:** 10.1186/s12889-021-11066-3

**Published:** 2021-06-05

**Authors:** K. Janeckova, Z. Hamrik, M. Matusova, P. Badura

**Affiliations:** Department of Recreation and Leisure Studies, Faculty of Physical Culture, Palacky University Olomouc, Tr. Miru 115, 771 11 Olomouc, Czech Republic

**Keywords:** Self-organized activities, Unstructured leisure, Sedentary behaviour, Youth, Inactivity, Lifestyle

## Abstract

**Background:**

Lifestyle sport activities (e.g. parkour or skateboarding) are considered attractive and beneficial for a long-term commitment to physical activity (PA) and might be a great opportunity for adolescents who do not feel comfortable in an organized or competitive atmosphere. The purpose of the study was to assess whether participation in lifestyle activities is associated with moderate-to-vigorous physical activity (MVPA), out-of-school vigorous physical activity (VPA), and sedentary behaviour in adolescents aged 10–15 years, with major demographic variables (sex, age, socioeconomic status) being taken into account.

**Methods:**

Data from a research project linked to the Health Behaviour in School-aged Children (HBSC) survey collected in 2017 in the Czech Republic was used. The sample consisted of 679 participants (303 of them girls) and was selected by quota sampling. Chi-square tests were used to assess differences in involvement in lifestyle activities according to sex, grade, and socioeconomic status. Ordinal and linear regression models were used to analyse the associations of participation in lifestyle activities and selected energy balance-related behaviours.

**Results:**

Participation in lifestyle sport activities was significantly associated with a higher level of physical activity (MVPA and out-of-school VPA) after adjustment for sociodemographic factors, as was participation in organized sport. No significant associations were shown for sedentary behaviour.

**Conclusions:**

Adolescents participating in lifestyle sport activities report being more physically active and, in case of doing multiple such activities concurrently, also spending less time sitting than their peers not involved in lifestyle sport activities. As such, lifestyle sport activities seem to represent a feasible way of increasing overall PA level in adolescent population.

**Supplementary Information:**

The online version contains supplementary material available at 10.1186/s12889-021-11066-3.

## Highlights

Participation in lifestyle sport activities might be a feasible way and one that appeals to young people to combat the decline in, or even lead to an increase in, the overall physical activity level in adolescents.

## Introduction

Lifestyle sport activities (LSA) have a place in contemporary society as a modern part of youth sport culture and represent a wide range of leisure-time activities in the out-of-school time [1]. LSA, such as workouts, slackline, parkour, free running, or skateboarding, are characterized as usually unsupervised, peer-oriented activities similar to free play where young people coordinate themselves during their leisure time [2]. They were traditionally classified as unstructured or unorganized [3]. However, some authors suggest labelling them as self-organized activities since neither of the two terms captures the nature of these activities, in spite of the lack of strict rules, formal leaders, or a priori performance goals typical of unstructured or unorganized activities. Unlike in unstructured activities (e.g. hanging out or partying), the absence of these features when it comes to rules or adult supervision leads to the development of a sense of autonomy and the power of will as the key competence [4].

Previous findings suggest that participation in unstructured leisure-time activities in young people is often associated with problematic outcomes, including poorer psychological adjustment [5] or engagement in health-risk [6] or antisocial behaviours [7]. These unfavourable outcomes, however, were related to activities such as hanging out with friends, spending time at shopping malls, or even partying. Another stream of studies showed numerous benefits of unstructured leisure-time activities for the positive development of young people. Unstructured activities, including LSA (e.g. skateboarding, hiking, or fishing) were associated with a lower rate of antisocial behaviour, better physical performance, psychological adjustment, connection with positive peer networks, and the building of one’s youth identity [3, 8–11]. This implies that it is important to differentiate between unstructured activities in order to help tackle a public discourse misinterpretation of lifestyle sports as a deviant activity [4]. Given these facts and taking into consideration the fact that physical inactivity in adolescence is a widely recognized serious public health issue [12], it is appropriate to focus on the promotion of sport activities that might be interesting for adolescents, which also concerns the previously mentioned self-organized LSA.

Physical activity (PA) is crucial for one’s health and healthy development during youth [13]. Nevertheless, studies show that PA decreases during adolescence [11] and international guidelines are not met globally among adolescents [12]. However, the new WHO PA guidelines emphasize that ‘every move counts’ indicating that PA at suboptimal levels is also beneficial [14]. Another factor affecting health is the amount of sedentary behaviour (sitting during leisure time), which may contribute to unhealthy consequences independently of that of regular PA [15].

LSA are considered attractive and beneficial for a long-term commitment to PA [1] and may act as a potential vehicle to increase levels of PA among adolescents. Self-organized LSA are related to the quality of infrastructure, can usually be done free of charge and might provide a great opportunity for adolescents who do not feel comfortable in an organized or competitive atmosphere related to sport and, thus, support their motivation for PA [16]. Moreover, some studies highlight the phenomenon that adolescents with a lower socioeconomic status (SES) engage less in organized sports [17]. LSA might not be affected by that to the same extent, since skateboarding, parkour, or workouts can be perceived as low-cost alternatives. It is, however, not yet clear how social status may affect participation in LSA, and therefore future studies are recommended.

To the best of our knowledge, there are only a limited number of studies aimed at investigating the relationships between self-organized LSA and PA or inactivity. Thus, the main aim of our study is to assess whether participation in LSA is associated with moderate-to-vigorous PA, out-of-school vigorous PA, organized sports, and sedentary behaviour in adolescents aged 10–15 years. Furthermore, the authors aim to assess whether the major demographic variables moderate these associations.

## Methods

### Procedures

The data for the study was drawn from a project titled “Development and validation of a questionnaire exploring leisure activities for the population of adolescents aged 10-15 years”. The project was conducted as a part of the activities of the Leisure Conceptual Group within the Health Behaviour in School-aged Children (HBSC) study.

The data collection took place in the autumn (September–October) of 2017 in seven ‘basic schools’ in the Olomouc region in the Czech Republic. In the Czech Republic, the usual basic school provides compulsory education from Grade 1 (mean age 6–7 years) to Grade 9 (mean age 14–15 years), which covers International Standard Classification of Education (ISCED) levels 1 and 2 [18]*.* The schools were located at seven different sites, with their selection being based on quota sampling to account for the administrative units in the region and size of the municipality. One was from a city (over 100,000 inhabitants), three from smaller towns (under 50,000 inhabitants), and three from villages (less than 1000 inhabitants). The data was collected through paper-based questionnaires by trained research administrators during regular class time. To avoid the risk of potential response bias, teachers were not present in the classroom at the time of the survey.

The study was approved by the Ethics Committee of Palacky University Olomouc. Participation in the survey was anonymous and voluntary; informed consent for participation in the questionnaire survey from the adolescent’s parents or guardians was required.

### Participants

Out of the 1017 pupils registered in the selected schools, a total number of 856 adolescents (an 84% response rate) completed the questionnaire. One hundred sixty-one adolescents were not present at school during the data collection – 62 were absent through illness, 27 were missing for another reason, and 72 refused to complete the questionnaire or did not provide informed consent. One hundred seventy-seven questionnaires were deleted because of incomplete answers or a lack of credibility (e.g. contradictory responses, repetitive response patterns, vulgarisms in open questions). We assessed the differences in demographic characteristics between the participants included in the analyses and those who were excluded using chi-square tests*.* There were no significant differences between the male and female adolescents (*p* = 0.506) or in relation to the size of the municipality (*p* = 0.526) and SES (*p =* 0.584). Nevertheless, there were significantly more missing values among the fifth-graders (65% of all missing values for LSA variables), compared with the older respondents. A single questionnaire version was used for all the age categories, with the questions on LSA being located in its second half. Younger respondents usually need more time to fill in their responses, compared to their older mates, which explains the higher rate of missing values among the fifth-graders. In total, data from 679 adolescents was analysed: 303 girls; 199 from the fifth grade, 248 from the seventh grade, and 232 from the ninth grade. The mean age of the fifth-graders was 10.7 years (±0.45). Among the seventh-graders it was 12.8 years (±0.43) and the mean age of the ninth-graders was 14.8 years (±0.49).

### Survey items

See Additional File 1 for the questionnaire items exploring leisure activities for the population of adolescents aged 10–15 years.

### Lifestyle sport activities (LSA)

The independent variable of main interest, participation in LSA (represented by workouts; parkour/free running; skateboarding/longboarding/penny boarding, etc.; freestyle scootering/skating, BMX (bicycle motocross), etc.), was measured using the question “How often do you engage in any of the following so-called lifestyle sport activities?” The respondents were asked to check one of four possible responses for each LSA: I don’t do this type of activity; a few times a year at most; about once or twice a month; and once a week or more often. The responses were categorized into three groups: 1) no LSA – adolescents who do not participate in any LSA on a regular (weekly) basis; 2) one LSA – those adolescents who do at least one of the mentioned LSA once a week or more often; and 3) two or more LSA – those adolescents who do two or more LSA at least once a week*.* LSA is an ‘in-house’ developed question for the HBSC leisure questionnaire. Results from a validation study (*N* = 616) comprising focus groups with adolescents and the test-retest procedure indicated the good stability of the responses over time. The LSA item has acceptable test-retest reliability (ICC = 0.62) for the total sum of mentioned lifestyle sport activities which adolescents do at least once a week or more often. Further information is available from the authors upon request.

Energy balance-related behaviours:

#### 1) Moderate-to-vigorous physical activity (MVPA)

Next, the participants were asked about their daily physical activity for a total of at least 60 min a day by the question: “Over the past seven days, on how many days were you physically active for a total of at least 60 minutes per day?” The responses ranged from 0 to 7 days (the values from zero to seven used for linear regression correspond to the number of days) [19]. It was indicated that the MVPA item had a reasonable validity [20] and it was shown to have acceptable test-retest reliability in adolescents from three Central European countries (ICC = 0.60) [21].

#### 2) Out-of-school vigorous physical activity (VPA)

Out-of-school VPA was measured by asking: “Outside school hours, how often do you usually exercise in your free time so much that you get out of breath or sweat?” with possible responses never = 1; less than once a month = 2 once a month = 3; once a week = 4; two to three times a week = 5; four to six times a week = 6; every day = 7. For ordinal regression analysis the whole scale was used. Similarly to MVPA, the out-of-school VPA item also has acceptable test-retest reliability (ICC = 0.62) [21] and its sufficient criterion validity was shown against fitness levels [22].

#### 3) Sedentary behaviour (SB)

SB was measured by a single item: “In your free time, which of the following statements best describes your typical sedentary habits?” The respondents chose from the following five statements: I spend almost none of my free time sitting = 1; I spend little time sitting during my free time = 2; I spend a moderate amount of my free time sitting = 3; I spend a lot of my free time sitting = 4; I spend almost all of my free time sitting = 5. For ordinal regression analysis the whole scale was used. This SB item was adopted from the 15-item Youth Activity Profile (YAP) and validated in a US sample, with the sedentary items of the YAP showing strong correlation with accelerometer-derived sitting time estimates [23].

Covariates:

### Organized sports (OS)

Participation in organized sport was assessed by the question: “In your leisure time, how often do you do any of the following organized activities?”, which was followed by an explanatory text: Organized activities are understood as activities performed in a sports club or a different club or organization under the leadership of a coach, teacher, instructor, or leader. The activities included team sports (e.g. football, volleyball, floorball) and individual sports (e.g., tennis, gymnastics, karate) [24]. The participants could choose from four answers (I don’t do this activity; once or twice per month; once per week; twice or more times per week). Both the activities served as control variables in the regression analyses. The organized activities scale has acceptable test-retest reliability (ICC = 0.64) [25].

### Socioeconomic status (SES)

The Family Affluence Scale III (FAS) was used to estimate the respondents’ SES. It contained six questions: Does your family own a car or another motor vehicle?; Do you have your own bedroom?; How many computers does your family own?; How many bathrooms are there in your home?; Does your family have a dishwasher?; How many times did you and your family travel out of the Czech Republic for a holiday last year? For descriptive purposes, the sum of all responses was split into three categories indicating (0–6) low affluence, (7–9) medium affluence, and (10–13) high affluence, as in a recent study from the Czech Republic [26]. Otherwise, we treated FAS as a continuous adjusting variable in the regression analyses. FAS is an index developed for the purposes of the HBSC study as a suitable and age-appropriate indicator of the SES of adolescents’ families [27]. In the Czech Republic, its validity showed a positive correlation between the FAS index and regional disposable income (*r* = 0.77, *p* < 0.01) [28].

### Statistical analysis

First, the composition of the study population was described (Table 1). This included the rates of the respondents’ involvement in LSA and organized sport activities for the total sample and also after stratification by sex, grade, and SES. The statistical significance of differences in involvement by sex, grade, and SES was estimated by the chi-square test. Spearman correlation analysis was performed in order to determine the relationship between participation in organized sports and LSA. The sociodemographic differences in energy balance-related behaviours, as dependent variables, were tested using Student’s t-test and ANOVA (Table 2).

**Table 1:** Description of the study population: rates of respondents’ involvement in lifestyle sport activities and organized sports, by sex, grade, and socioeconomic status; Czech Republic, 2017

	Total	Sex			Grades				SES			
	(*n* = 679)	Boys (*n* = 376)	Girls (*n* = 303)	*p*-value	5. (*n* = 199)	7. (*n* = 248)	9. (*n* = 232)	*p*-value	Low (*n* = 149)	Medium (*n* = 279)	High (*n* = 154)	*p*-value
No LSA	437(64.4%)	219 (58.2%)	**218 (71.9%)**	**< 0.001**	129 (64.8%)	163 (65.7%)	145 (62.5%)	ns	98 (65.8%)	184 (65.9%)	94 (61.0%)	ns
LSA 1	168 (24.7%)	**103 (27.4%)**	65 (21.5%)	**< 0.001**	45 (22.6%)	58 (23.4%)	65 (28%)	ns	39 (26.2%)	64 (22.9%)	36 (23.4%)	ns
LSA 2+	74 (10.9%)	**54 (14.4%)**	20 (6.6%)	**< 0.001**	25 (12.6%)	27 (10.9%)	22 (9.5%)	ns	12 (8.1%)	31 (11.1%)	24 (15.6%)	ns
Playground Workout	89 (13.1%)	**72 (19.1%)**	17 (5.6%)	**< 0.001**	9 (4.5%)	26 (10.5%)	**54 (23.3%)**	**< 0.001**	16 (10.7%)	43 (15.4%)	25 (16.2%)	ns
Parkour/free running	69 (10.2%)	**53 (14.1%)**	16 (5.3%)	**< 0.001**	**31 (15.6%)**	22 (8.9%)	16 (6.9%)	**< 0.01**	12 (8.1%)	24 (8.6%)	19 (12.3%)	ns
Skateboarding etc.	106 (15.6%)	51 (13.6%)	55 (18.2%)	ns	32 (16.1%)	45 (18.1%)	29 (12.5%)	ns	19 (12.8%)	44 (15.8%)	29 (18.8%)	ns
Freeride scooter/BMX etc.	78 (11.5%)	**56 (14.9%)**	22 (7.3%)	**< 0.01**	**31 (15.6%)**	29 (11.7%)	18 (7.8%)	**< 0.05**	20 (13.4%)	24 (8.6%)	24 (15.6%)	ns
OS individual	120 (17.7%)	60 (16%)	60 (19.8%)	ns	**43 (21.6%)**	32 (12.9%)	45 (19.4%)	**< 0.05**	14 (9.4%)	50 (17.9%)	**41 (26.6%)**	**< 0.001**
OS team	248 (36.5%)	**177 (47.1%)**	71 (23.4%)	**< 0.001**	73 (36.7%)	91 (36.7%)	84 (36.2%)	ns	46 (30.9%)	108 (38.7%)	63 (40.9%)	ns
OS total	331 (48.7%)	**216 (57.4%)**	115 (38%)	**< 0.001**	107 (53.8%)	110 (44.4%)	114 (49.1%)	ns	57 (38.3%)	143 (51.3%)	**90 (58.4%)**	**< 0.01**

% represents the relative rate of valid responses; statistically significant (*p* < 0.05) differences according to sex, grade, or SES are in bold; SES socioeconomic status measured by family affluence scale; No LSA adolescents who do not participate in any LSA on a regular (weekly) basis; LSA 1 lifestyle sport activities (at least one activity once a week or more often); LSA 2+ lifestyle sport activities (two activities or more once a week or more often); OS organized sport (two or more times per week)

**Table 2:** Energy balance-related behaviours by sex, grade, and socioeconomic status; Czech Republic, 2017

			***N***	***Mean (Sd)***	***df***	***F/t***	***Effect size***	***p***
**Sex**	MVPA	boys	376	4.05 (±2.11)	677	3.03	0.234	**0.003**
		girls	303	3.59 (±1.81)				
	VPA	boys	376	4.9 (±1.62)		3.16	0.244	**0.002**
		girls	303	4.51 (±1.59)				
	SB	boys	376	2.63 (±0.95)		−1.18	−0.091	0.238
		girls	303	2.73 (±0.93)				
**Grade**	MVPA	5. grade	199	3.75(±2.04)	2	0.42	0.001	0.654
		7. grade	248	3.84 (±2.05)				
		9. grade	232	3.93 (±1.90)				
	VPA	5. grade	199	4.9 (±1.61)		1.67	0.005	0.189
		7. grade	248	4.63 (±1.59)				
		9. grade	232	4.69 (±1.65)				
	SB	5. grade	199	2.59 (±0.97)		1.84	0.005	0.159
		7. grade	248	2.65 (±0.88)				
		9. grade	232	2.76 (±0.98)				
**SES**	MVPA	low	149	3.58 (±2.12)	2	3.54	0.012	**0.030**
		medium	279	3.73 (±1.85)				
		high	154	4.15 (±1.99)				
	VPA	low	149	4.56 (±1.81)		2.84	0.010	0.059
		medium	279	4.70 (±1.61)				
		high	154	4.99 (±1.45)				
	SB	low	149	2.76 (±0.98)		2.03	0.007	0.132
		medium	279	2.65 (±0.94)				
		high	154	2.54 (±0.92)				

MVPA moderate-to-vigorous physical activity; (the values 0–7 used correspond to numbers days per week when the respondents were physically active for at least 60 min)

VPA out-of-school vigorous physical activity; 1 = never; 2 = less than once a month; 3 = once a month; 4 = once a week; 5 = 2 to 3 times a week; 6 = 4 to 6 times a week; 7 = every day; SB sedentary behaviour; 1 = I spend almost none of my free time sitting; 2 = I spend little time sitting during my free time; 3 = I spend a moderate amount of my free time sitting; 4 = I spend a lot of my free time sitting; 5 = I spend almost all of my free time sitting; SES socioeconomic status; *df* degree of freedom; *f*: small effect 0.1; medium 0.25; large 0.4; Effect size: small 0.01; medium 0.059; large 0.138; *p* < 0.05; *p* < 0.01; *p* < 0.001 statistical significance in bold

Next, the associations of LSA with energy balance-related behaviours were assessed using linear regression (MVPA) and ordinal regression (out-of-school VPA, SB). The authors started with the crude Model 1 (Table 3). Next, the analysis was adjusted for sex, grade, and socioeconomic status (Model 2), and lastly also for organized sports (Model 3). The stability of the results was also tested by including the interaction effects of participation in LSA with sex, grade, and socioeconomic status. All of the data was analysed using the programs jamovi (version 1.6) and IBM SPSS (IBM Corp. Released 2013. Armonk, NY: IBM Corp) version 22 for Windows.

**Table 3:** Associations of adolescents’ involvement in lifestyle sport activities with physical activity and sedentary behaviour; Czech Republic, 2017

	MVPA *β* (95% CI)	VPA OR (95% CI)	SB OR (95% CI)
**MODEL 1** (*univariate)*			
No LSA	*Ref.*	*Ref.*	*Ref.*
LSA 1	0.75*** (0.38–1.12)	1.93*** (1.36–2.73)	0.89 (0.62–1.27)
LSA 2+	1.37*** (0.87–1.87)	2.48*** (1.55–3.99)	0.37*** (0.23–0.60)
**MODEL 2** (*adjusted for sex, grade and SES)*			
No LSA	*Ref.*	*Ref.*	*Ref.*
LSA 1	0.69*** (0.32–1.06)	1.88*** (1.33–2.67)	0.87 (0.60–1.25)
LSA 2+	1.25*** (0.75–1.75)	2.13** (1.31–3.45)	0.41*** (0.25–0.67)
**MODEL 3** (*adjusted for sex, grades, SES, organized sport)*			
No LSA	*Ref.*	*Ref.*	*Ref.*
LSA 1	0.56** (0.20–0.92)	1.75** (1.23–2.50)	0.93 (0.64–1.35)
LSA 2+	1.13*** (0.65–1.61)	2.27** (1.38–3.76)	0.40*** (0.24–0.66)

*LSA* lifestyle sport activity; *SES* socioeconomic status; *MVPA* moderate-to-vigorous physical activity

*VPA* out-of-school vigorous physical activity; *SB* sedentary behaviour

**p < 0.01; ***p < 0.001

## Results

**Fig. 1:**
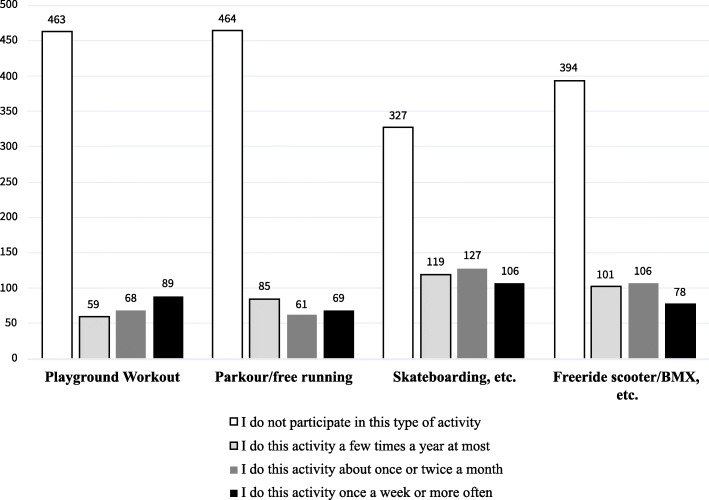
The frequency of participation in specific lifestyle sport activities (*N*=679)

Table 1 describes involvement in LSA and organized sports by sex, grade, and SES. Out of the 679 respondents, slightly over a third of the school-aged adolescents were involved in LSA on at least a weekly basis. A quarter (24.7%) of the sample reported engaging in one LSA at least once a week, and 10.9% in two or more such activities at least weekly. The frequency of participation for specific LSA is displayed in Fig. 1.

Skateboarding, longboarding, penny boarding, etc., were the most prevalent types of LSA, with 15.6% of our sample engaging in this activity on a weekly basis. However, the differences from participation in other LSA were negligible: playground workouts (13.1%), parkour and free running (10.2%), and freestyle scooter, skating, BMX, etc. (11.5%). Overall, the authors observed a higher number of boys than girls (*p* < 0.001) participating in LSA. The same held true for playground workouts (*p* < 0.001), parkour and free running (*p* < 0.001), and freestyle scooter, skating, BMX, etc. (*p* < 0.01), but not for skateboarding and penny boarding. The boys also reported a significantly higher frequency of participation in organized team sports than the girls (*p* < 0.001).

The data analyses showed that 108 respondents participated in LSA without being involved in any organized sports and 134 respondents participated in LSA and concurrently in one or more organized sport activities. The results of the correlation test showed that there was a significant yet weak relationship (*r*_*s*_ = 0.099, *p* < 0.01) between LSA and organized sports among the school-aged adolescents.

Regarding age categories, a higher number of the ninth-graders (i.e. 14–15-year-olds) engaged in playground workouts (*p* < 0.001), compared with their younger peers. Conversely, fifth-graders (i.e. 10–11-year-olds) reported parkour and free running (*p* < 0.01) and freestyle scooter, skating, BMX, etc. (*p* < 0.05) more frequently than the older respondents.

No SES-related differences were observed for overall participation in LSA or in specific LSA. The only significant result with regard to SES was found for organized individual sports, with a higher rate of individual sport participants being shown in the higher-SES group (*p* < 0.001).

Table 2 presents the results of the one-way ANOVA test and Student’s t-test assessing the differences in energy balance-related behaviours according to sociodemographic factors. The boys reported higher levels of MVPA and out-of-school VPA than the girls. We did not observe any differences in MVPA, out-of-school VPA, or SB by grade. Lastly, the MVPA levels differed according to SES, with those who were more affluent also reporting higher levels of MVPA compared to their less affluent peers.

Table 3 presents the associations of participation in LSA and three selected energy balance-related behaviours in adolescents. Involvement in one LSA showed significant associations with MVPA, as well as out-of-school VPA, both in the crude and adjusted models (*β* = 0.56; 95% CI 0.20–0.92 for MVPA; OR = 1.75; 95% CI = 1.23–2.50 for out-of-school VPA). Even stronger associations were observed for involvement in two or more LSA (*β* = 1.13; 95% CI = 0.65–1.61 for MVPA; OR = 2.27; 95% CI = 1.38–3.76 for out-of-school VPA). We did not find any significant association between engaging in one LSA and SB but those who engaged in two or more LSA were more likely to spend less leisure time sitting (OR = 0.40; 95% CI = 0.24–0.66) than their counterparts who were not involved in LSA at all. Assessing the interaction effects of the association of participation in LSA with MVPA, out-of-school VPA, and SB regarding sex, grade, and socioeconomic status showed results that were not statistically significant.

## Discussion

The main aim of the study was to assess whether participation in LSA was associated with energy balance-related behaviours in adolescents aged 10–15 years. Furthermore, the authors assessed whether the major demographic variables moderated this association. The findings showed that participation in LSA was associated with higher involvement in physical activity (MVPA and out-of-school VPA). The association was already observable for one LSA done at least weekly, but was even stronger for more than one LSA. This also held true after controlling for sex, grade, and socioeconomic status, as well as participation in organized sport. Such information may support the theory that lifestyle sports may increase the level of PA among adolescents or at least contribute to compliance with the PA guidelines [4, 12]. Apart from the physiological response to movement itself during LSA, the authors assume LSA are advantageous and may also increase PA because adolescents already use their bikes or skates to travel to skate parks, workout playgrounds, etc.

Another factor of lifestyle is sedentary behaviour, which may have a negative effect on health independently of PA [15]. The authors hypothesized that participation in LSA may be associated with less time spent sitting. This was confirmed only in those adolescents who reported at least two LSA on a weekly basis or more often. Our results are in line with previous studies that indicated similar patterns in adolescents partaking in outdoor play. The minimum of 1 day of outdoor PA may reduce the total amount of SB among adolescents [29] and may also contribute in terms of prevention of adolescent obesity [30].

The boys engaged in LSA more often than the girls did. This finding is in accordance with a study reporting males engaging in self-organized PA more than females [16]. Similar patterns of sex disparity can be seen in overall PA levels in the Czech Republic and boys, in general, also tend to be more physically active than girls in other European countries [31]. This would imply that LSA, analogously to many other types of PA, are more attractive for boys. An alternative explanation could be that equally sex-balanced LSA were not used in the questionnaire. LSA which are preferred by girls, such as street dance, breakdance, or hip-hop, were missing. These are labelled as recreational dance activities often dominated by women [32].

The results of the survey showed that 35.6% of the school-aged adolescents engaged in LSA at least once a week or more. Taking into account the above-mentioned results, this finding should be considered by municipal policymakers. In comparison with ‘traditional’ sports, these sorts of activities (and the related infrastructure) have only been partly systematically supported in the Czech Republic to a sufficient extent thus far. Given the youth-friendly nature of LSA, which is apparent from the fact that nearly one-third of the sample reported engaging in them, the development of an ‘LSA-friendly’ environment (construction and maintenance of skateparks, workout playgrounds, etc.) could bring social benefits [1] and could potentially promote the level of PA in adolescents. This could apply especially to those who do not feel comfortable in organized and competitive settings [33]. The perceptions of task climate were associated with adaptive intrinsic forms of motivational regulation in a systematic review of the intrapersonal correlates of motivational climate perceptions in sport and PA [34]. It is possible that adherence to LSA could be stronger in some portion of the population than adherence to organized sports, which would in turn also contribute to adopting a lifelong commitment to PA. On the other hand, we must acknowledge the possibility that LSA would only displace other types of PA, but this clearly warrants further research.

The proportion of participants practising parkour, free running, freestyle scooter, skating, BMX, and individual OS decreased slightly with age. Adolescents may drop out from these activities because they feel that they are ‘not good enough’ when compared with other peers [35]. Our findings are in line with the specialization theory [36]. The theory proposes that for adolescents aged around 13 years, enjoyment and social interactions through play and the development of skill in specific sports through practice are the basic characteristics of their involvement in sport. Adolescents spend their leisure time more with peers and so become more independent of their families. LSA as self-organized activities are similar by their nature to OS. The difference could be seen in the sense that having fun instead of focusing deeply on competitive success could therefore reduce the drop-out rate from LSA and support the inner motivation of adolescents and thus contribute to changing the patterns of how they spend their leisure time and create active lifelong habits [33].

Lastly, in extreme situations such as the ongoing COVID-19 pandemic, when the lockdown in many countries all over the world has resulted in school closures and a ban on organized activities [37], it is assumed that LSA, with their specific self-organized nature, may provide an opportunity for adolescents to maintain their level of PA by encouraging time spent outdoors [38]. Some recent studies also offer recommendations for home-based physical activity during the pandemic [39] and there would be space to complement them with LSA.

### Strengths and limitations

The current study provides valuable insights into lifestyle sports concerning energy balance-related behaviours in adolescence, which seem to be a relevant area of interest [4] that provides researchers and policymakers with a more holistic understanding of how adolescents get used to doing physical activity. However, there are some limitations of this study. This study is cross-sectional and, thus, it is impossible to infer causality. The data was collected through paper-based questionnaires. Even though the authors used a valid international standardized tool for assessment of PA, working with self-reported data can represent the risk of possible real distortion of facts. Nevertheless, the survey items are also used in wider international studies and have shown satisfactory validity and reliability [21, 40]. Lastly, the data was collected only in a single region of the Czech Republic, fifth-graders were underrepresented in the sample and thus, the results are hardly generalizable to the entire adolescent population, distinct age groups, or other geographical areas.

## Conclusions

Participation in LSA in adolescence is associated with a higher level of involvement in out-of-school vigorous physical activity and moderate-to-vigorous physical activity and with less time spent sitting when participating in two or more LSA. This finding also holds true after accounting for the effects of sex, grade, SES, and participation in organized sport. Given that participation in LSA was reported by approximately a third of the adolescents in this study, it appears that these sorts of activities could be useful for promoting PA levels from this age category at the population level. Investigators might extend the research regarding LSA and regional, age, and gender-specific patterns to help develop strategies and policies promoting adolescents’ physical activity.

## Supplementary Information

**Additional file 1.** Questionnaire exploring leisure activities for the population of adolescents aged 10–15 years.

## Data Availability

The datasets used and/or analysed during the current study are available from the corresponding author on reasonable request.

## Abbreviations

BMX: bicycle motocross
CI: confidence interval
FAS: Family Affluence Scale
HBSC: Health Behaviour in School-aged Children
LSA: lifestyle sport activities
MVPA: moderate-to-vigorous physical activity
OR: odds ratios
OS: organized sports
PA: physical activity
SB: sedentary behaviour
SES: socioeconomic status
SPSS: Statistical Package for the Social Sciences
VPA: out-of-school vigorous physical activity
WHO: World Health Organization

## References

[1] Gilchrist P, Wheaton B (2017). The social benefits of informal and lifestyle sports: a research agenda. Int J Sport Policy..

[2] Osgood DW, Anderson AL, Shaffer JN (2005). Unstructured leisure in the after-school hours. Organ Act As Context Dev Extracurricular Act After Sch Community Programs.

[3] Bradley GL (2010). Skate parks as a context for adolescent development. J Adolesc Res.

[4] Säfvenbom R, Wheaton B, Agans JP (2018). ‘How can you enjoy sports if you are under control by others?’ Self-organized lifestyle sports and youth development. Sport Soc.

[5] Persson A, Kerr M, Stattin H (2007). Staying in or moving away from structured activities: explanations involving parents and peers. Dev Psychol.

[6] Badura P, Madarasova Geckova A, Sigmundova D, Sigmund E, van Dijk JP, Reijneveld SA (2018). Can organized leisure-time activities buffer the negative outcomes of unstructured activities for adolescents’ health?. Int J Public Health..

[7] McClelland C, Giles AR (2014). Street-involved youth’s unstructured leisure: activities and their social consequences. Leis Loisir.

[8] Bignold WJ (2013). Developing school students’ identity and engagement through lifestyle sports: a case study of unicycling. Sport Educ Soc.

[9] Gilchrist P, Osborn G (2017). Risk and benefits in lifestyle sports: parkour, law and social value. Int J Sport Policy.

[10] Gilchrist P, Wheaton B (2011). Lifestyle sport, public policy and youth engagement: examining the emergence of parkour. Int J Sport Policy..

[11] Corder K, Sharp SJ, Atkin AJ, Griffin SJ, Jones AP, Ekelund U, van Sluijs EMF (2015). Change in objectively measured physical activity during the transition to adolescence. Br J Sports Med.

[12] Sallis JF, Bull F, Guthold R, Heath GW, Inoue S, Kelly P, Oyeyemi AL, Perez LG, Richards J, Hallal PC (2016). Progress in physical activity over the Olympic quadrennium. Lancet..

[13] Warburton DER, Bredin SSD (2017). Health benefits of physical activity: a systematic review of current systematic reviews. Curr Opin Cardiol.

[14] World Health Organization (2020). WHO guidelines on physical activity and sedentary behaviour.

[15] Väistö J, Eloranta AM, Viitasalo A, Tompuri T, Lintu N, Karjalainen P, Lampinen EK, Ågren J, Laaksonen DE, Lakka HM, Lindi V. Physical activity and sedentary behaviour in relation to cardiometabolic risk in children: cross-sectional findings from the physical activity and nutrition in children (PANIC) study. Int J Behav Nutr Phys Act 2014;11(1):1–10. 10.1186/1479-5868-11-55. 10.1186/1479-5868-11-55 PMC4008488 24766669

[16] Wiium N, Säfvenbom R (2019). Participation in organized sports and self-organized physical activity: associations with developmental factors. Int J Environ Res Public Health.

[17] Pot N, Verbeek J, van der Zwan J, van Hilvoorde I (2016). Socialisation into organized sports of young adolescents with a lower socio-economic status. Sport Educ Soc.

[18] Schneider SL (2013). The international standard classification of education 2011. Comp Soc Res.

[19] Prochaska JJ, Sallis JF, Long B (2001). A physical activity screening measure for use with adolescents in primary care. Arch Pediatr Adolesc Med.

[20] Biddle SJH, Gorely T, Pearson N, Bull FC (2011). An assessment of self-reported physical activity instruments in young people for population surveillance: project ALPHA. Int J Behav Nutr Phys Act.

[21] Bobakova D, Hamrik Z, Badura P, Sigmundova D, Nalecz H, Kalman M (2014). Test–retest reliability of selected physical activity and sedentary behaviour HBSC items in the Czech Republic, Slovakia and Poland. Int J Public Health..

[22] Booth ML, Okely AD, Chey T, Bauman A (2001). The reliability and validity of the physical activity questions in the WHO health behaviour in schoolchildren (HBSC) survey: a population study. Br J Sports Med.

[23] Saint-Maurice PF, Welk GJ (2015). Validity and calibration of the youth activity profile. PLoS One.

[24] Badura P, Geckova AM, Sigmundova D, Van Dijk JP, Reijneveld SA (2015). When children play, they feel better: organized activity participation and health in adolescents energy balance-related behaviors. BMC Public Health.

[25] Bosakova L, Kolarcik P, Bobakova D, Sulcova M, Van Dijk JP, Reijneveld SA (2016). Test–retest reliability of the scale of participation in organized activities among adolescents in the Czech Republic and Slovakia. Int J Public Health.

[26] Sigmund E, Sigmundová D, Badura P, Voráčová J, Vladimír H, Hollein T (2020). Time-trends and correlates of obesity in Czech adolescents in relation to family socioeconomic status over a 16-year study period (2002-2018). BMC Public Health.

[27] Hartley JEK, Levin K, Currie C (2016). A new version of the HBSC family affluence scale - FAS III: Scottish qualitative findings from the international FAS development study. Child Indic Res.

[28] Hobza V, Hamrik Z, Bucksch J, De Clercq B. The family affluence scale as an indicator for socioeconomic status: Validation on regional income differences in the Czech Republic. Int J Environ Res Public Health. 2017;14(12):1–9. 10.3390/ijerph14121540 PMC5750958 29292773

[29] Sampasa-Kanyinga H, Colman I, Hamilton HA, Chaput JP (2020). Outdoor physical activity, compliance with the physical activity, screen time, and sleep duration recommendations, and excess weight among adolescents. Obes Sci Pract.

[30] Zhang Y, Zhang X, Li J, Zhong H, Pan CW (2020). Associations of outdoor activity and screen time with adiposity: findings from rural Chinese adolescents with relatively low adiposity risks. BMC Public Health.

[31] Inchley J, Currie D, Budisavljevic S, Torsheim T, Jåstad A, Cosma A, et al. World Health Organization. Regional Office for Europe. Spotlight on adolescent health and well-being. Findings from the 2017/2018 Health Behaviour in School-aged Children ( HBSC) survey in Europe and Canada. International report. 2020;2:25–30.

[32] Markula P (2020). Dance, movement and leisure cultures. Leis Stud.

[33] Witt PA, Dangi TB (2018). Why children/youth drop out of sports. J Park Recreat Admi.

[34] Harwood CG, Keegan RJ, Smith JMJ, Raine AS (2015). A systematic review of the intrapersonal correlates of motivational climate perceptions in sport and physical activity. Psychol Sport Exerc.

[35] Vandell DL, Larson RW, Mahoney JL, Watts TW. Children’s organized activities. Handb Child Psychol Dev Sci. 2015;4(7):1–40.

[36] Coté J, Hay J (2002). Children’ s involvement in sport : a developmental perspective. Psychol Found Sport.

[37] Chen P, Mao L, Nassis GP, Harmer P, Ainsworth BE, Li F (2020). Coronavirus disease (COVID-19): the need to maintain regular physical activity while taking precautions. J Sport Heal Sci.

[38] Riazi NA, Wunderlich K, Gierc M, Brussoni M, Moore SA, Tremblay MS, et al. “ You Can’t Go to the Park, You Can’t Go Here, You Can’t Go There”: Exploring Parental Experiences of COVID - 19 and Its Impact on Their Children’s Movement Behaviours. 2021;8(3):219. 10.3390/children8030219 PMC8000735 33809221

[39] Ricci F, Izzicupo P, Moscucci F, Sciomer S, Maffei S, Di Baldassarre A, Gallina S (2020). Recommendations for physical inactivity and sedentary behavior during the coronavirus disease (COVID-19) pandemic. Front Public Health.

[40] Liu Y, Wang M, Tynjälä J, Lv Y, Villberg J, Zhang Z (2010). Test-retest reliability of selected items of health behaviour in school-aged children (HBSC) survey questionnaire in Beijing, China. BMC Med Res Methodol.

