# Supplementary material for: “I am going out!” – lifestyle sports and physical activity in adolescents
Source: BMC Public Health. 2021 Jun 5;21:1079. doi: 10.1186/s12889-021-11066-3 (PMC8179071; doi:10.1186/s12889-021-11066-3)
Supplement: Supplementary file 1 — Additional file 1. Questionnaire exploring leisure activities for the population of adolescents aged 10–15 years. [file 12889_2021_11066_MOESM1_ESM.docx]

**Additional File 1.**  **Questionnaire exploring leisure activities for the population of adolescents aged** **10-15 years**

***Demographic Information***

| 1. **Are you a girl or a boy?** | | | | | |
| --- | --- | --- | --- | --- | --- |
| (1) | 🞎 | Boy | (2) | 🞎 | Girl |

| 1. **What class are you in?** | | | |
| --- | --- | --- | --- |
| (1) | 🞎 | 5^th^ grade |  |
| (2) | 🞎 | 7^th^ grade |  |
| (3) | 🞎 | 9^th^ grade |  |
| (4) | 🞎 | Other grade, please specify: …………………………………………………… | |

| 1. **What month were you born?** | | | | | | | | | | | |
| --- | --- | --- | --- | --- | --- | --- | --- | --- | --- | --- | --- |
| Jan | Feb | Mar | Apr | May | June | July | Aug | Sep | Oct | Nov | Dec |
| 🞎 | 🞎 | 🞎 | 🞎 | 🞎 | 🞎 | 🞎 | 🞎 | 🞎 | 🞎 | 🞎 | 🞎 |

| 1. **What year were you born?** | | | | | | | | | |
| --- | --- | --- | --- | --- | --- | --- | --- | --- | --- |
| 1999 | 2000 | 2001 | 2002 | 2003 | 2004 | 2005 | 2006 | 2007 | 2008 |
| 🞎 | 🞎 | 🞎 | 🞎 | 🞎 | 🞎 | 🞎 | 🞎 | 🞎 | 🞎 |

| 1. **How much do you weigh without clothes?** | ………………………… **kg** |
| --- | --- |
| 1. **How tall are you without shoes?** | ………………………… **cm** |

1. **How many people live in the city/village where you live?**

| (1) | 🞎 | 50 000 inhabitants or more |
| --- | --- | --- |
| (2) | 🞎 | 10 000 - 49 999 inhabitants |
| (3) | 🞎 | 2 000 - 9 999 inhabitants |
| (4) | 🞎 | Less than 2 000 inhabitants |

***Physical activity*** *is any activity that increases your heart rate and makes you get out of breath some of the time. Physical activity can be done in sports, school activities, playing with friends, or walking to school. Some examples of physical activity are running, brisk walking, rollerblading, biking, dancing, skateboarding, swimming, soccer, basketball, football, skiing & surfing.*

1. **Over the past 7 days, on how many days were you physically active for a total of at least 60 minutes per day?**

*Please add up all the time you spent in physical activity each day.*

| 0 days | 1 day | 2 days | 3 days | 4 days | 5 days | 6 days | 7 days |
| --- | --- | --- | --- | --- | --- | --- | --- |
| 🞎 | 🞎 | 🞎 | 🞎 | 🞎 | 🞎 | 🞎 | 🞎 |

The following questions relate to leisure time. **Leisure time** should be understood as time you have for yourself when you don’t have to do any homework or housework. Leisure time is your free time that you can spend freely; you can, for example, do your hobbies (sport, drawing, reading, and many others). These activities are collectively called **leisure activities**

1. **In your leisure time, how often do you do any of the following organized activities? Organized activities should be understood as activities performed in a sports club or a different club or organization under the leadership of a coach, teacher, instructor, or leader. DO NOT INCLUDE activities such as when you and your friends occasionally play football or go to the gym.**

*Please tick one box in each row.*

|  | (1) | (2) | (3) | (4) |
| --- | --- | --- | --- | --- |
|  | I don’t do this type of activity | About once or twice a month | Once a week | Twice a week or more |
| 1. Organized team sports (for example football, volleyball, floorball) | 🞎 | 🞎 | 🞎 | 🞎 |
| 1. Organized individual sports (for example tennis, gymnastics, karate) | 🞎 | 🞎 | 🞎 | 🞎 |
| 1. Artistic activities (for example art and music school, playing a musical instrument, singing, dancing, drama) | 🞎 | 🞎 | 🞎 | 🞎 |
| 1. Children’s and youth organizations (for example Scouting, Sokol, Junák, YMCA) | 🞎 | 🞎 | 🞎 | 🞎 |
| 1. Club in a leisure centre or at school (for example board games, model-making, language or debating club) | 🞎 | 🞎 | 🞎 | 🞎 |
| 1. Religious activities (for example church meetings, singing in a church choir) | 🞎 | 🞎 | 🞎 | 🞎 |
| 1. Volunteering (for example nature protection, walking shelter dogs, helping in a hospice) | 🞎 | 🞎 | 🞎 | 🞎 |
| 1. Political organizations (for example Young Conservatives, Young Social Democrats) | 🞎 | 🞎 | 🞎 | 🞎 |

1. **Outside school hours: how often do you usually exercise in your free time so much that you get out of breath or sweat?**

| (1) | 🞎 | Every day |
| --- | --- | --- |
| (2) | 🞎 | 4-6× a week |
| (3) | 🞎 | 3× a week |
| (4) | 🞎 | 2× a week |
| (5) | 🞎 | 1× a week |
| (6) | 🞎 | 1× a month |
| (7) | 🞎 | Less than once a month |
| (8) | 🞎 | Never |

1. **Which of the following best describes your typical sedentary habits at home? (Try to think about a typical week and not just last week)**

| (1) | 🞎 | I spend almost none of my free time sitting |
| --- | --- | --- |
| (2) | 🞎 | I spend little time sitting during my free time |
| (3) | 🞎 | I spend a moderate amount of time sitting during my free time |
| (4) | 🞎 | I spend a lot of time sitting during my free time |
| (5) | 🞎 | I spend almost all of my free time sitting |

1. **How often do you engage in any of the following, so-called lifestyle sport activities?**

*Please tick one box in each row.*

|  | (1) | (2) | (3) | (4) |
| --- | --- | --- | --- | --- |
|  | I don’t do this type of activity | A few times a year at most | About once or twice a month | Once a week or more |
| 1. Workout | 🞎 | 🞎 | 🞎 | 🞎 |
| 1. Slackline | 🞎 | 🞎 | 🞎 | 🞎 |
| 1. Parkour, free running | 🞎 | 🞎 | 🞎 | 🞎 |
| 1. Footbag (hacky sack) | 🞎 | 🞎 | 🞎 | 🞎 |
| 1. Juggling, flowersticks, yoyo etc. | 🞎 | 🞎 | 🞎 | 🞎 |
| 1. Skateboarding, longboarding, penny board etc. | 🞎 | 🞎 | 🞎 | 🞎 |
| 1. Freestyle BMX, freestyle scootering, freestyle in-line etc. | 🞎 | 🞎 | 🞎 | 🞎 |
| 1. Indoor climbing | 🞎 | 🞎 | 🞎 | 🞎 |
| 1. Other activity… Please specify?............ | 🞎 | 🞎 | 🞎 | 🞎 |

| 1. **Does your family own a car, van or truck?** | | |
| --- | --- | --- |
| (1) | 🞎 | No |
| (2) | 🞎 | Yes, one |
| (3) | 🞎 | Yes, two or more |

| 1. **Do you have your own bedroom for yourself?** | | | | | |
| --- | --- | --- | --- | --- | --- |
| (1) | 🞎 | No | (2) | 🞎 | Yes |

| 1. **How many computers do your family own (including laptops and tablets, not including game consoles and smartphones)?** | | |
| --- | --- | --- |
| (1) | 🞎 | None |
| (2) | 🞎 | One |
| (3) | 🞎 | Two |
| (4) | 🞎 | More than two |

| 1. **Does your family have a dishwasher at home?** | | | | | |
| --- | --- | --- | --- | --- | --- |
| (1) | 🞎 | No | (2) | 🞎 | Yes |

| 1. **How many bathrooms (room with a bath/shower or both) are in your home?** | | |
| --- | --- | --- |
| (1) | 🞎 | None |
| (2) | 🞎 | One |
| (3) | 🞎 | Two |
| (4) | 🞎 | More than two |

1. **In the last year, How many time were you and your family on vacation …**

*Please tick one box in each row.*

|  | (1) | (2) | (3) | (4) |
| --- | --- | --- | --- | --- |
|  | Not at all | Once | Twice | More than twice |
| 1. … **outside** the Czech Republic? | 🞎 | 🞎 | 🞎 | 🞎 |
| 1. … **in** the Czech Republic? | 🞎 | 🞎 | 🞎 | 🞎 |
